# Effects of Vinorelbine on M2 Macrophages in Non-Small Cell Lung Cancer

**DOI:** 10.3390/ijms26052252

**Published:** 2025-03-03

**Authors:** Ahmed Al-Omar, Milad Asadi, Ufuk Mert, Can Muftuoglu, Haydar Soydaner Karakus, Tuncay Goksel, Ayse Caner

**Affiliations:** 1 Department of Basic Oncology, Institute of Health Sciences, Ege University, Bornova 35100, Izmir, Turkey; ahmedalomar2024@gmail.com (A.A.-O.); miladasadi1389@gmail.com (M.A.); canmuftuoglu1990@gmail.com (C.M.); 2 Atatürk Health Care Vocational School, Ege University, Bornova 35100, Izmir, Turkey; ufuk.mert@ege.edu.tr; 3 Translational Pulmonary Research Group (EGESAM), Ege University, Bornova 35100, Izmir, Turkey; haydar.soydaner.karakus@ege.edu.tr (H.S.K.); tuncay.goksel@ege.edu.tr (T.G.); 4 Department of Pulmonary Medicine, Faculty of Medicine, Ege University, Bornova 35100, Izmir, Turkey; 5 Department of Parasitology, Faculty of Medicine, Ege University, Bornova 35100, Izmir, Turkey

**Keywords:** non-small-cell lung cancer, vinorelbine, tumor-associated macrophage, polarization, M2 macrophage phenotype

## Abstract

Tumor-associated macrophages (TAMs) significantly influence tumor progression and patient responses to conventional chemotherapy. However, the interplay between anti-cancer drugs, immune responses in the tumor microenvironment, and their implications for cancer treatment remains poorly understood. This study investigates the effects of vinorelbine on M2 macrophages in lung cancer and its capacity to modulate TAMs toward an M1 phenotype. Peripheral blood mononuclear cells (PBMCs) were polarized into M2 macrophages, and subsequent phenotype alterations upon vinorelbine treatment were assessed. Additionally, we evaluated vinorelbine’s impact on gene and protein expression associated with cancer progression and cell invasion in non-small-cell lung cancer (NSCLC) cells indirectly co-cultured with M2 macrophages. Notably, vinorelbine, particularly at low concentrations, reprogrammed M2 macrophages to exhibit M1-like characteristics. While M2 macrophages enhanced cancer cell invasion, vinorelbine significantly mitigated this effect. M2 macrophages led to the overexpression of numerous genes linked to tumor growth, angiogenesis, invasion, and immune suppression in NSCLC cells, increasing the BCL2/BAX ratio and promoting cellular resistance to apoptosis. The anti-tumor efficacy of vinorelbine appears to be partly attributed to the reprogramming of M2 macrophages to the M1 phenotype, suggesting that low-dose vinorelbine may optimize therapeutic outcomes while minimizing toxicity in cancer patients.

## 1. Introduction

Non-small-cell lung cancer (NSCLC) patients have high mortality rates due to the aggressiveness of cancer and early-stage metastasis [1]. According to Globocan data, the incidence of lung cancer in both sexes is estimated to be 11.4% and the mortality rate is 18% in 2020 [2]. Lung cancer is generally divided into two subtypes; non-small-cell lung cancer (NSCLC), which accounts for approximately 80–85%, and small-cell lung cancer, which accounts for 15–20% [3]. The treatment regimen for NSCLC is mainly based on the stage of cancer and includes surgery, radiotherapy, immunotherapy, chemotherapy, and targeted therapy [4]. Some combination treatment modalities are also used, such as the combination of chemotherapies and immunotherapies as first-line therapy [5]. Although most chemotherapy regimens primarily affect only lung neoplastic cells, some can also target cells in the tumor microenvironment (TME), such as tumor-associated macrophages (TAMs) [6].

Macrophages are unique components of both innate and adaptive immunity and eliminate tumor cells, while M2-like TAMs participate in the formation of the tumor microenvironment, promoting tumor growth, invasion, metastasis, and drug resistance [7,8,9]. Generally, there are two main phenotypes of macrophages that can be polarized depending on biological stimuli and environments [8]. M1 macrophages are classically activated and are considered to have an anti-tumor role by scavenging phagocytosed tumor cells and stimulating an inflammatory response [9]. The M2 phenotype consists of alternatively activated macrophages with opposite functions such as anti-inflammatory responses, tissue remodeling, angiogenic regulation, tumor cell proliferation, and metastasis [9]. Monocytes circulating in the bloodstream differentiate into macrophages when they reach the TME, and then they may be polarized into M1- or M2-like TAMs depending on stimulating factors secreted by cancer and stromal cells [10]. In the TME, M1 macrophages can limit lung cancer progression; however, M2 TAMs can cause tumor cell growth and enhance epithelial–mesenchymal transition (EMT), thereby resulting in poor patient outcomes [7,8]. TAMs promote the development of metastasis by supporting the formation of all five steps (invasion, intravasation, survival in circulation, extravasation, and progressive growth) necessary for cancer cells to metastasize [11]. TAMs can also suppress other immune cells by secreting cytokines, growth factors, and chemokines and triggering the inhibitory immune checkpoints on T cells [11].

Therapeutic strategies based on or combined with macrophages may play an important role in increasing the efficacy of anti-cancer therapies. There are many strategies to target M2-like TAMs, one of which depends on targeting cancer cells and M2-like TAMs. Studies have shown that integrating therapeutic strategies targeting TAMs with conventional treatments yields promising results [11,12,13]. Other strategies are to repolarize TAMs to the M1 phenotype and inhibit macrophage recruitment [11]. Ultimately, therapeutics targeting TAMs and polarizing TAMs towards M1 are expected to achieve favorable clinical effects, especially in combination with chemotherapeutics.

Vinorelbine, which is used in lung cancer treatment, is a compound belonging to the family of vinca alkaloids. The cytotoxic effects of vinorelbine against tumor cells are mediated through the inhibition of microtubule formation and cell cycle arrest [14]. It causes mitotic shift through retention of the cell in the G2 phase and inhibition of cytokinesis. Subsequently, the occurrence of multinucleation triggers DNA damage, resulting in mitotic apoptosis [12]. It was reported that NSCLC patients with stage II and IIIA cancer and receiving cisplatin and vinorelbine had higher survival rates compared to cisplatin combined with chemotherapy. However, no significant difference was found in patients with stage IA or IB [13]. Recently, studies have revealed that the use of vinorelbine as a metronomic chemotherapy shows efficacy, reduced toxicity, and improved quality of life. Metronomic oral vinorelbine is now an option for the first- or later-line treatment of selected individuals with advanced breast cancer or NSCLC [15].

**Figure 1:**
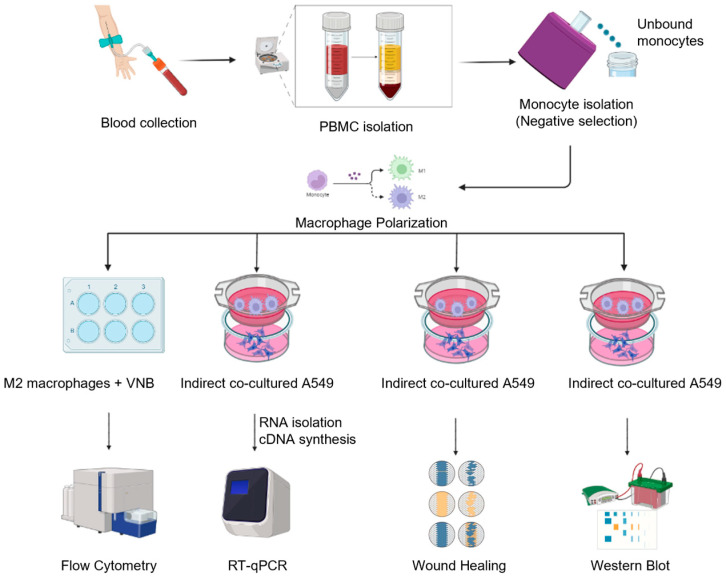
The schematics represent a summary of the experimental methods.

The use of anti-cancer agents that can directly block the expression of aggressive factors related to cancer malignancy and indirectly remodulate M2 macrophages can enhance the anti-cancer response. The aim of this study is to investigate the ability of vinorelbine to remodulate M2-like TAMs into M1 macrophages and the effects of vinorelbine on the progression and metastasis of NSCLC cells co-cultured with M2 macrophages. The schematic design of the experimental methods is shown in Figure 1.

## 2. Results

### 2.1. Vinorelbine Inhibits the Proliferation of NSCLC

**Figure 2:**
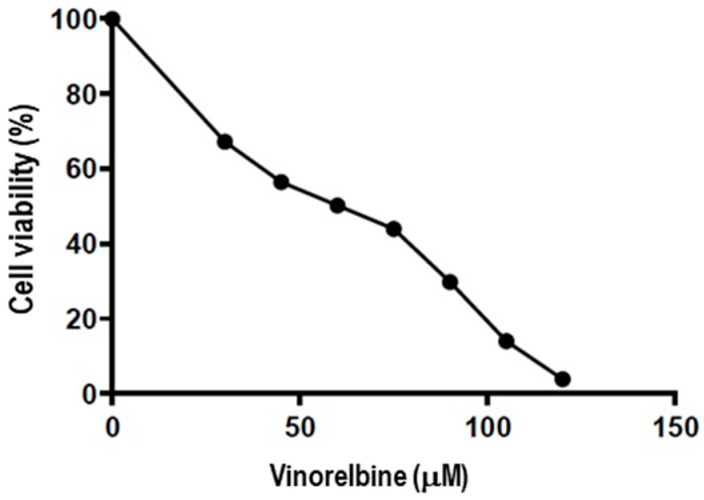
The cytotoxic efficacy of vinorelbine on A549 cells. Cancer cells were treated with different concentrations of (0–120 μM) of vinorelbine for 24 h. Cell viability was assessed by an XTT assay. The IC50 of vinorelbine was calculated using GraphPad 7 software.

The cytotoxicity of vinorelbine on A549 cells was determined using an XTT assay. Vinorelbine inhibited cell proliferation in a dose-dependent manner. The analyzed data showed that the half-maximal inhibitory concentration (IC50) of vinorelbine in A549 cells was 50.13 μM (Figure 2).

### 2.2. Macrophage Polarization into M1 and M2 Phenotypes

**Figure 3:**
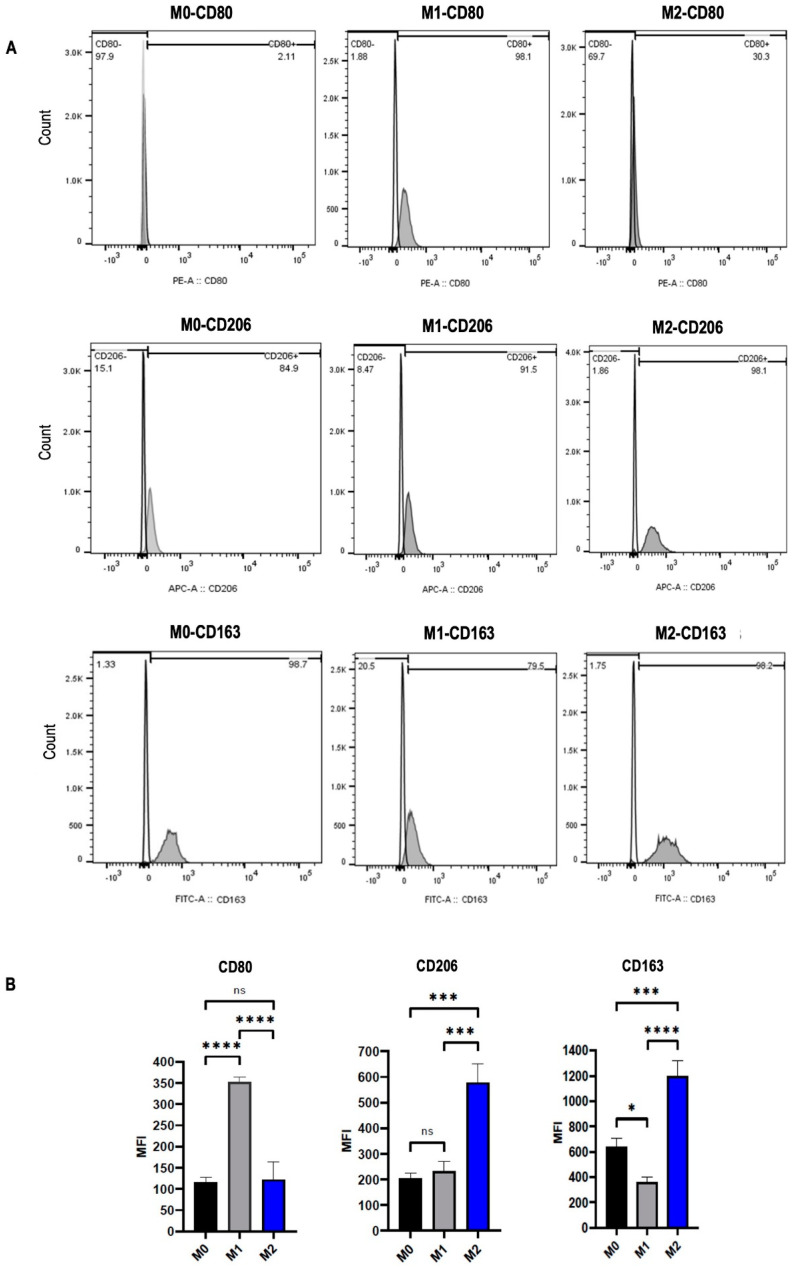
Polarization of human monocyte-derived macrophages (MDMs). Primary MDMs (M0) were unstimulated or were polarized into M1 (20 ng/mL LPS) and M2 (25 ng/mL IL-4 plus 25 ng/mL IL-10) for 48 h. Surface marker expression was analyzed by flow cytometry. (**A**) Histograms of percentages and (**B**) Boxplot graphs of MFI values for CD80, CD206, and CD163 after stimulating with LPS or IL-4/IL-10 for 48 h. The groups are compared with the control and with each other. Statistical analyses were performed using ANOVA, * *p* < 0.05; *** *p* < 0.001; **** *p* < 0.0001, ns; nonsignificant.

To polarize M0 macrophages into M1 and M2 subtypes, monocytes isolated from peripheral blood were differentiated into M0 macrophages through incubation with M-CSF. M0 cells treated with LPS were differentiated into M1 macrophages, while those treated with IL-4 plus IL-10 were differentiated into M2 macrophages. Then, all M0, M1, and M2 macrophages were stained with anti-CD80, anti-CD206, and anti-CD163 antibodies for flow cytometry analysis. CD80, which is a specific marker for M1 macrophages, was significantly upregulated after induction with LPS (*p* < 0.0001). In macrophages stimulated with IL-4 plus IL-10, CD206, and CD163, which are specific markers for M2 macrophages, were remarkably increased in the M2 subtype compared to M0 (*p* < 0.001 and *p* < 0.001, respectively) and M1 (*p* < 0.001 and *p* < 0.0001, respectively) (Figure 3B). The results indicated that the stimulatory factors polarized macrophages into M1 and M2 subtypes.

### 2.3. Vinorelbine Modulates M2 Macrophages into the M1 Phenotype

**Figure 4:**
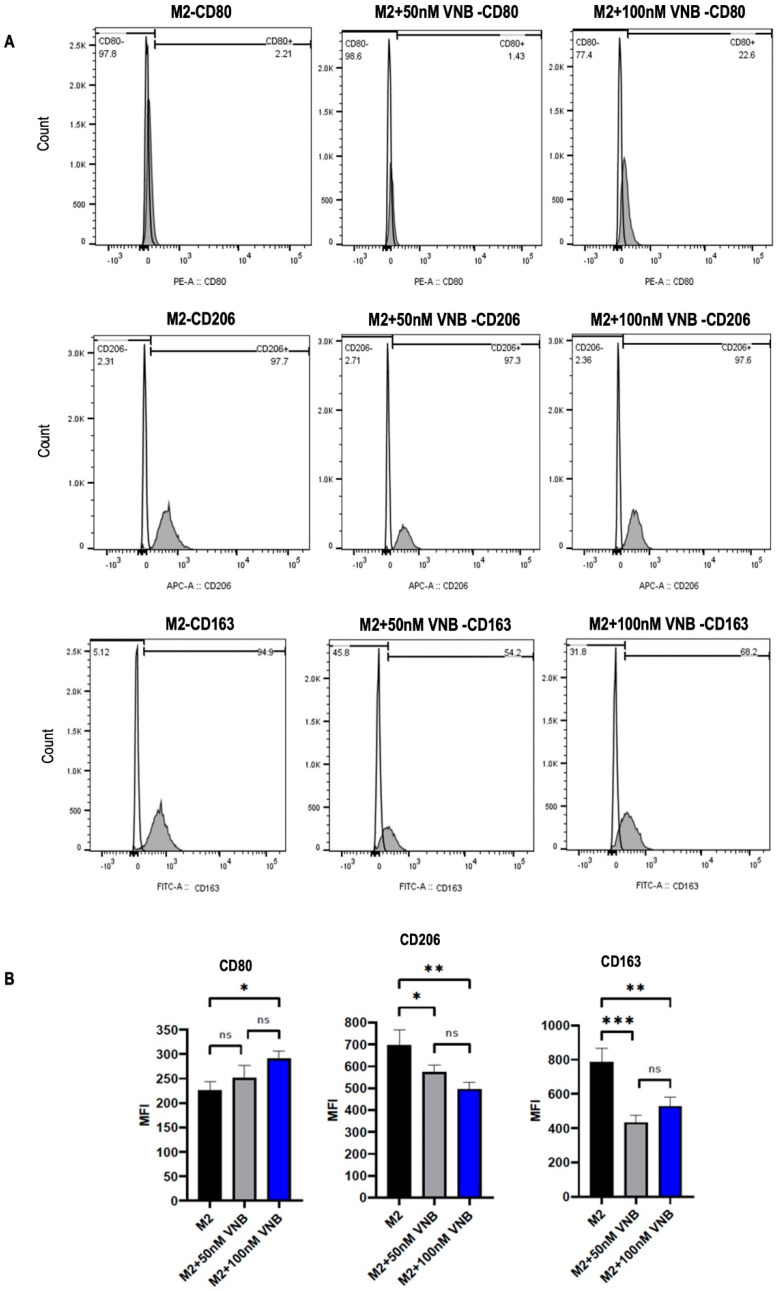
Vinorelbine reprogrammed M2 macrophages into the M1 subtype. Primary MDMs (M0) were polarized into M2 (25 ng/mL IL-4 plus 25 ng/mL IL-10) for 48 h. Surface marker expression was analyzed by flow cytometry. (**A**) Histograms of percentages and (**B**) Boxplot graphs of MFI values for CD80, CD206, and CD163 after treatment of M2 macrophages with/without vinorelbine (50 nM or 100 nM) for 48 h. The groups were compared with the control and with each other. VNB: Vinorelbine. Statistical analyses were performed with ANOVA, * *p* < 0.05; ** *p* < 0.01; *** *p* < 0.001; ns; nonsignificant.

To investigate the efficacy of vinorelbine on M2 TAMs, the monocytes were polarized into M2 macrophages. Then, the cells were treated with two different doses of vinorelbine (50 nM and 100 nM) for 48 h and analyzed by flow cytometry. A control group of untreated vinorelbine M2 macrophages was also analyzed at the same time. Compared to the control group, the expression of CD80 was significantly increased in the group treated with 100 nM vinorelbine (*p* < 0.05), while there was a non-significant increase in the group treated with 50 nM vinorelbine. On the other hand, the expression of CD206 was significantly reduced in groups treated with 50 nM and 100 nM vinorelbine in a dose-dependent manner (*p* < 0.05 and *p* < 0.01, respectively). Similarly, the expression of CD163 was markedly decreased in groups treated with 50 nM and 100 nM vinorelbine (*p* < 0.001 and *p* < 0.01, respectively) (Figure 4B). The results suggest that vinorelbine has the potential to remodulate M2 macrophages into the M1 phenotype.

### 2.4. The Effect of M2 Macrophages and Vinorelbine on Gene Expression Levels of A549 Cells

**Figure 5:**
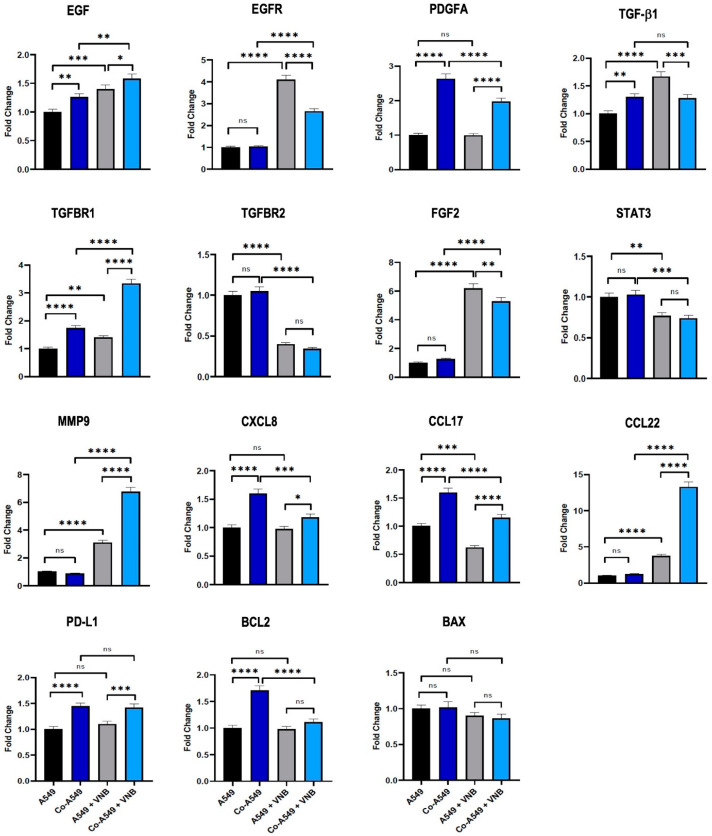
Vinorelbine increased mRNA expression levels of many genes that enhance cancer progression in NSCLC cells. A549 and A549 cells co-cultured with M2 macrophages were treated with vinorelbine (50 mM) for 24 h. Cells were harvested for RNA extraction, and mRNA expression levels of the genes were quantified by RT-qPCR. The groups were compared with the control and with each other. Statistical analyses were performed with ANOVA, * *p* < 0.05; ** *p* < 0.01; *** *p* < 0.001; **** *p* < 0.0001, ns; nonsignificant. VNB: Vinorelbine; Co-A549: co-cultured A549 cells. The colors of the boxes in the graph are shown as black; A549, dark blue; Co-A549, Grey; A549 + VNB, light-blue; Co-A549 + VNB.

Increasing evidence has revealed that many factors secreted and produced by TAMs enhance cancer progression, such as EGF, PDGF, CXCL8, MMP9, and FGF2 in the TME. Furthermore, some cytokines, such as CCL17, CCL22, and TGF-b, cause direct or indirect immunosuppression [9,16]. To evaluate the effect of M2 macrophages on vinorelbine, expression levels of genes associated with cancer progression were measured in A549 cells. The results showed that the expression levels of EGF, PDGFA, TGF-b1, TGFBR1, CXCL8, CCL17, PD-L1, and BCL2 were significantly increased in A549 cells co-cultured with M2 macrophages compared to the control group. In A549 cells treated with vinorelbine, the expression levels of TGFBR2, STAT3, and CCL17 were significantly reduced, while the expression levels of EGF, EGFR, TGF-b1, TGFBR1, FGF2, MMP9, and CCL22 were significantly upregulated. In co-cultured A549 cells treated with vinorelbine, the expression levels of EGF, TGFBR1, MMP9, and CCL22 were more upregulated than in other groups. On the other hand, M2 macrophages affected the regulation of some genes (EGFR, PDGFA, FGF2, CXCL8, and CCL17) in A549 cells, leading to a decrease in the efficacy of vinorelbine (Figure 5). The results showed that despite the anti-tumoral efficacy of vinorelbine, the drug increases the expression of some malignant genes in A549 cells, similar to how M2 macrophages decrease the anti-tumoral efficacy of vinorelbine.

### 2.5. The Effects of M2 Macrophages and Vinorelbine on A549 Cells at the Protein Level

The effect of M2 macrophages and vinorelbine on proteins that are involved in lung cancer progression was evaluated using Western blotting. Vinorelbine upregulated the expression levels of EGFR and phosphorylated EGFR (p-EGFR) proteins in A549 cells after 24 h and 48 h compared to the untreated control cells. In cancer cells co-cultured with M2, while p-EGFR was upregulated compared to A549 cells, EGFR expression levels were almost the same. Interestingly, vinorelbine led to more upregulation of EGFR and p-EGFR in co-cultured cells than in untreated co-cultured cells. In addition, vinorelbine did not affect BAX expression in cancer cells at 24 h but decreased it at 48 h. On the other hand, protein expression levels of BAX in co-cultured cells treated with/without vinorelbine were downregulated compared to the control group.

**Figure 6:**
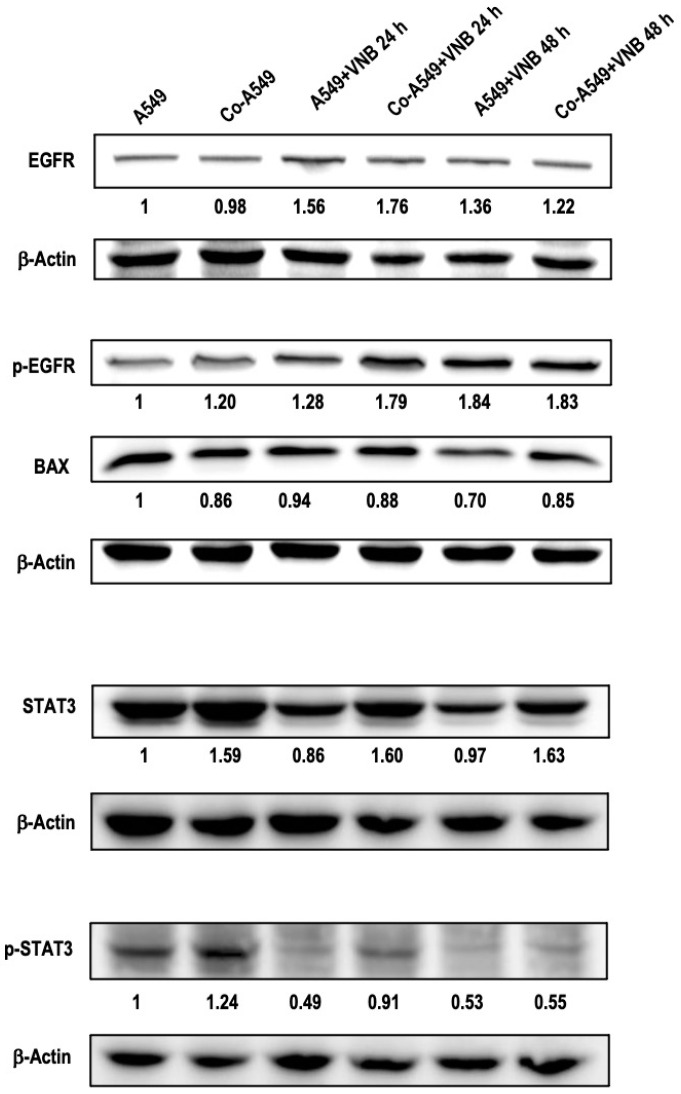
Vinorelbine upregulated p-EGFR and downregulated p-STAT3 in NSCLC cells. A549 and co-cultured A549 cells were treated with vinorelbine (50 mM) for 24 h and 48 h. Cell lysates were collected and the protein expression levels of EGFR, p-EGFR, BAX, STAT3, and p-STAT3 were evaluated by Western blotting. B-actin was used as a loading control. Protein bands were analyzed using Image J software version 1.53 and the values below indicate relative expression levels compared with the control. VNB: Vinorelbine; Co-A549: co-cultured A549 cells.

Vinorelbine led to greater downregulation of p-STAT3 expression than STAT3 expression in cancer cells at 24 h. The decrease in p-STAT3 expression stayed remarkable at 48 h, while there was no change in STAT3 expression at 48 h compared to control cells. In addition, STAT3 and p-STAT3 were upregulated in co-cultured cells. Vinorelbine remarkably downregulated p-STAT3 in a time-dependent manner while STAT3 expression did not change compared to co-cultured cells (Figure 6). M2 macrophages increased the expression levels of the activated form of malignant factors and reduced pro-apoptotic protein levels in cancer cells. In addition, M2 macrophages decreased the anti-tumor effects of vinorelbine by upregulating p-STAT3 and p-EGFR and downregulating BAX.

### 2.6. Vinorelbine Inhibits the Migration of NSCLC

**Figure 7:**
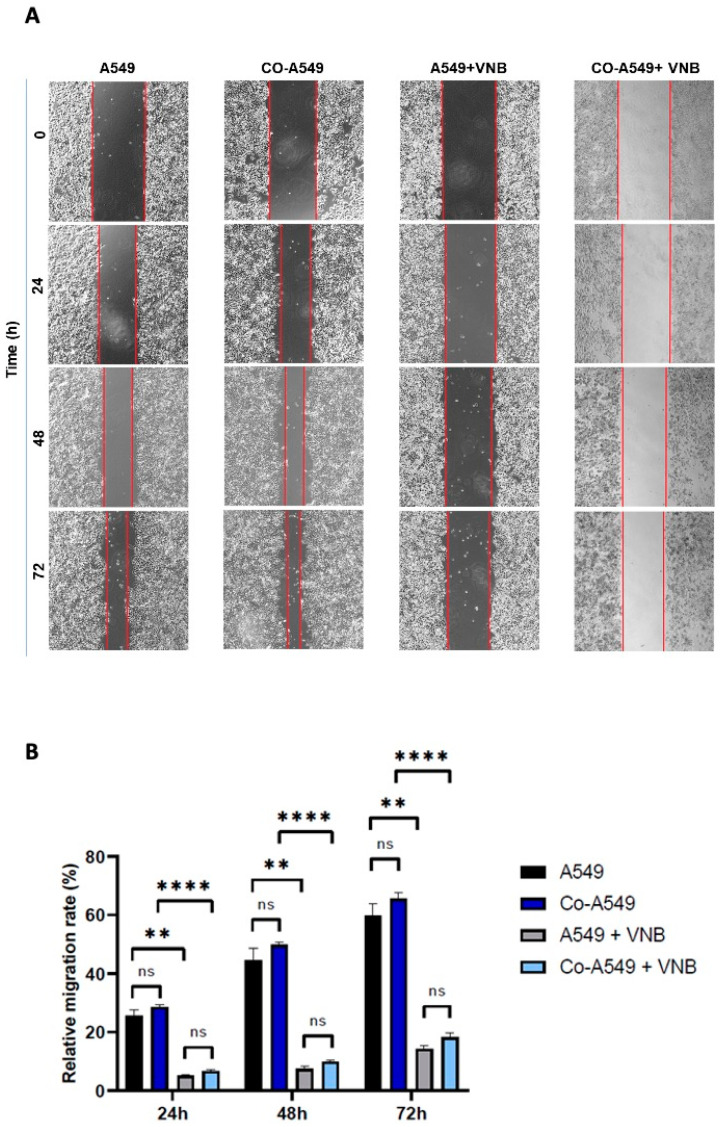
The efficacy of vinorelbine on non-small-cell lung cancer cell migration. (**A**) A549 or co-cultured A549 cells were scratched with a micropipette tip. After washing with PBS, vinorelbine-treated and untreated cells were incubated in medium. Images for different times (0 h, 24 h, 48 h, and 72 h) were taken. (**B**) Relative migration rate % of the cells, which is the percentage of healing of the scratch by the cells. The groups were compared with the control and with each other. Statistical analyses were performed with ANOVA, ** *p* < 0.01; **** *p* < 0.0001; ns = nonsignificant. VNB: Vinorelbine; Co-A549: co-cultured A549 cells.

To investigate the inhibitory efficacy of vinorelbine on the invasion and migration of cancer cells, a scratch wound model was used for 24, 48, and 72 h. The findings showed that the relative migration rate of control cells was 59.8% at 72 h, while co-cultured A549 cells migrated more rapidly, at a rate of 65.4%, but this increase was non-significant compared to control cells. After treatment with vinorelbine (20 μM) for 72 h, the relative migration rate of cancer cells was significantly decreased at 14.3% compared to control cells (*p* < 0.01). Similarly, the relative migration rate of co-cultured cells treated with vinorelbine also significantly decreased and was 18.2% at 72 h compared to non-treated co-cultured cells (*p* < 0.0001). Furthermore, in the groups treated with vinorelbine, the mobility of co-cultured cells was non-significantly higher than cancer cells. The rates of relative migration at 24 and 48 h were similar to those obtained at 72 h (Figure 7). The results indicated that vinorelbine effectively inhibits the migration of monocultured and co-cultured A549 cells, while M2 macrophages slightly increased the invasion of the cells and reduced the efficacy of vinorelbine on migration.

## 3. Discussion

Recent studies suggest that the therapeutic efficacy of several classical anti-cancer drugs is based on their capacity to alter the immune response in the tumor environment [17,18,19,20]. In this context, it may be important for treatment management to reveal that anti-cancer drugs have immunostimulant properties in addition to their antiproliferative effects [21,22]. Vinorelbine has long been used in medical oncology to treat NSCLC and breast cancer [15,23,24]. However, it is unknown how vinorelbine may alter the immune response to cancer in the tumor microenvironment, or how this microenvironment may affect the efficacy of the drug.

The activation state of macrophages is critical for balancing between tumor progression and suppression, providing a functional spectrum composed of M1 and M2 phenotypes [25]. Furthermore, macrophages have plasticity of repolarization from M1 into M2 macrophages and vice versa. Due to these properties, reprogramming M2-like TAMs towards anti-tumor M1 macrophages is a convenient strategy in cancer treatment [26]. Studies have shown that paclitaxel and gemcitabine used in cancer treatment can remodulate M2 macrophages into an M1 phenotype [6,27]. In another study, vinblastine, a vinca alkaloid and antineoplastic agent, has shown the ability to reprogram TAMs toward an M1-like phenotype. Following treatment with vinblastine for 24 h, there was a significant increase in the percentage of M1 marker expression. Furthermore, morphological observations revealed a transformation of the cells from an elongated shape characteristic of the M2 phenotype to a rounded shape indicative of the M1 phenotype [28]. In this study, we showed that vinorelbine reprogrammed immunosuppressive M2 macrophages into the M1 phenotype, especially at low concentrations. Particularly, metronomic vinorelbine is considered a treatment option in NSCLC patients due to its excellent safety profile and good balance between efficacy and tolerance [29]. Therefore, the effects of drugs at low concentrations on remodulating M2 macrophages into an M1-like phenotype provide an advantage for the metronomic approach. This finding may help explain why metronomic vinorelbine therapy is effective in patients with advanced NSCLC and high-risk populations. Most studies focus on investigating the effect of these antineoplastic agents on M2 macrophages because one of the ways that they achieve this reprogramming is by changing the mechanism of M2 to become the same as M1. For instance, one of the remodulating mechanisms is targeting the signaling pathways of M2 macrophages, such as STAT3/STAT6 inhibition or NF-κB activation, which is already blocked or active in M1 macrophages, respectively [28,30]. However, without further research, it is not possible to confirm that the drugs will not change or affect M1 macrophages. Therefore, future studies to investigate the interaction between drugs and the immune system are needed.

In addition, a previous study showed that vinorelbine inhibited the invasion and migration of lung, liver, and colon cancer cells [31]. Similarly, this study showed that vinorelbine significantly reduced the invasion of lung cancer cells. Particularly, although the M2 macrophages increased the invasion of cancer cells, vinorelbine significantly reduced this effect. On the other hand, M2 macrophages also reduced the efficacy of vinorelbine on lung cancer cells. On the other hand, M2 macrophages also reduced the invasion efficacy of vinorelbine on lung cancer cells.

TAM/M2 macrophages play an important role in tumor proliferation, invasion, angiogenesis, metastasis, and the immune suppression of the TME via secreting molecules (EGF, VEGF, PDGF, and FGF), cytokines (IL-10 and TGF-b), and chemokines (CCL17, CCL22, and CXCL8), as well as by inducing autocrine and paracrine pathways [32,33,34,35]. TAMs also produce proteolytic enzymes that mediate extracellular matrix (ECM) degradation, such as matrix metalloproteinases (MMP2, MMP7, and MMP9), serine proteases, and cathepsins, leading to metastasis [36]. In this way, TAM can promote genes or proteins involved in cancer cell growth and proliferation [16,33,37]. In this study, M2 macrophages induced the overexpression of many genes associated with tumor growth, angiogenesis, invasion, and immune suppression in A549 cells. In addition, M2 macrophages increased the BCL2/BAX ratio, making the cells more resistant to apoptosis.

Interestingly, our data suggest that vinorelbine upregulated the expression of genes that play important roles in cancer progression in A549 cells with/without co-culture. This efficacy of vinorelbine can be reduced, at least, by the metronomic approach in patients with lung cancer. Notably, the expression of p-EGFR was upregulated in A549 cells treated with vinorelbine and was even higher in co-cultured A549 cells treated with vinorelbine. These findings could be related to the degree of response of A549 cells to vinorelbine or the effect mechanism of vinorelbine. Studies have shown that A549 cells express the highest levels of EGFR compared to other NSCLC cell lines, such as H-292, H-358, and H-1975 [38,39]. A study also showed that A549 cells had the lowest sensitivity to vinorelbine compared to other cell lines of NSCLC [39]. Overexpression of EGFR in A549 cells could result in higher activation of AKT and ERK, which are considered a possible cause of vinorelbine treatment resistance [40]. Other NSCLC cell lines expressing low EGFR levels are more sensitive to vinorelbine due to the downregulation of certain factors such as MMP9 and BCL2 and the upregulation of BAX [31].

STAT3 signaling is involved in the immune regulation of macrophages and is essential for macrophage differentiation toward the M2 phenotype [41,42]. Activation of the transcription factor STAT3 also causes the production of different proteins and cytokines that regulate tumor growth, angiogenesis, metastasis, or resistance to anti-cancer therapies [43]. This study revealed that expression levels of STAT3 and p-STAT3 were significantly upregulated in cancer cells exposed to M2 macrophages, while they were significantly downregulated in cancer cells treated with vinorelbine. However, M2 macrophages reduced the efficacy of vinorelbine because p-STAT3 expression was less downregulated in co-cultured A549 cells. Similarly, this effect of M2 macrophages on vinorelbine was also detected in the expression of some genes, particularly those involved in metastasis, in cancer cells.

In conclusion, these findings suggest that the anti-tumor efficacy of vinorelbine is at least partially related to the reprogramming of M2 macrophages into the M1 phenotype. Furthermore, the efficacy of vinorelbine therapy against TAMs, at low doses, may biologically explain the therapy window of metronomic vinorelbine, which may protect cancer patients from unnecessary toxic treatments. This study should be validated by in vivo clinical trials addressing whether vinorelbine may repolarize M2 macrophages into M1-like macrophages or whether the effectiveness of vinorelbine varies with the amount of TAM in the TME. Therefore, further studies are needed to understand the potential immune effects of vinorelbine.

## 4. Materials and Methods

### 4.1. Cell Lines and Reagents

The human lung adenocarcinoma cell line A549 was kindly provided by the Department of Bioengineering (Ege University, Izmir, Turkey). Vinorelbine was obtained from the pharmacy as a solution of vinorelbine tartrate 50 mg/5 mL (Pierre Fabre, Istanbul, Turkey). The medium (RPMI 1640, with L-Glutamin), Ficoll, and lipopolysaccharides (LPS) were bought from Sigma-Aldrich (St. Louis, MO, USA). Fetal bovine serum (FBS), trypsin–EDTA, and TrypLE were purchased from GIBCO/BRL (Grand Island, NY, USA). Xpert Blue Cell Viability Assay (Grisp, Portugal). Macrophage colony-stimulating factor (M-CSF), interleukin-4 (IL-4), interleukin-10 (IL-10), and flow cytometry antibodies (CD206, CD80, and CD163) were provided by BioLegend (San Diego, CA, USA). Transwell 6-well plates and 12-well plates were obtained from Corning (Corning, NY, USA) and Sarstedt (Numbercht, Germany).

### 4.2. Cell Viability Assay

A549 cells were maintained in RPMI-1640 medium with L-glutamine supplemented with 10% fetal calf serum, 100 U/mL penicillin, and 100 µg/mL streptomycin at 37 °C in 5% CO_2_. A549 cells were cultured at a density of 1.5 × 10^4^ cells/well in 96-well culture plates for 24 h. Following this, the cells were treated with different concentrations of vinorelbine (30, 45, 60, 75, 90, 105, and 120 μM) for 24 h. Then, cell viability was determined using XTT (Grisp, Porto, Portugal) [40].

### 4.3. Cell Culture and Polarization of Macrophages

Human whole blood was obtained from different healthy donors at the Translational Pulmonary Research Center (EGESAM, Izmir, Turkey), and peripheral blood mononuclear cells (PBMCs) were isolated using Ficoll density gradient centrifugation. Monocytes were isolated from PBMCs using the EasySep Human Monocyte Isolation Kit (Stemcell Technologies, Vancouver, BC, Canada). For macrophage polarization, monocytes were cultured in RPMI-1640 medium supplemented with 10 % heat-inactivated serum, 1% penicillin–streptomycin (GIBCO/BRL Grand Island, NY, USA), and recombinant human rhM-CSF (50 ng/mL or 25 ng/mL) for 7 days. M0 cells were stimulated into M1 using LPS (20 ng/mL), and into M2 using rhIL-4 (25 ng/mL) plus rhIL-10 (25 ng/mL) for 2 days [28].

### 4.4. Flow Cytometry

Monocytes were first differentiated into M0 macrophages with M-CSF, and then M0 macrophages were polarized into M1 or M2 macrophages, respectively. For this, monocytes were seeded in 6-well plates (4.5 × 10^5^ cells/well) and incubated with M-CSF (25 ng/mL) for 7 days. After the monocytes were differentiated into M0 macrophages, M0 macrophages were stimulated with LPS or IL-4 (25 ng/mL) plus IL-10 (25 ng/mL) for 48 h and polarized into M1 or M2 macrophages, respectively. Then, three groups were formed for testing and the first group was incubated with free vinorelbine medium for 48 h. Simultaneously, the second and third groups were incubated with a medium containing vinorelbine 50 nM and 100 nM, respectively. Subsequently, cells were harvested and analyzed by flow cytometry with specific markers: CD80, CD206, and CD163 [28].

### 4.5. Indirect Co-Culture

Transwell inserts with a pore size of 0.4 mm on a 6-well plate were used for the indirect co-culture system. The isolated monocytes (4.5 × 10^5^ cell/transwell) were seeded on the upper chamber. The cells were treated with M-CSF (50 ng/mL) for 7 days to differentiate them into M0 macrophages. Then, the cells were stimulated with rhIL-4 (25 ng/mL) plus rhIL-10 (25 ng/mL) for 2 days on a different plate. On day 8, A549 cells (2.5 × 10^5^ cells/well) were seeded in the lower chamber and incubated for 24 h. Then, transwell inserts containing M2 macrophages were transferred onto A549 cells and incubated with serum-free conditions for 48 h. Monocultured lung cancer cells in serum-free conditions were used as the control [44].

### 4.6. Quantitative Real-Time Reverse-Transcription PCR

After A549 cells were treated with 50 μM of vinorelbine and incubated for 24 h, the total RNA was isolated from A549 cells using tripleXtractor (Grisp, Porto, Portugal) and converted to cDNA using a First Strand cDNA Synthesis Kit (New England Biolabs, Ipswich, MA, USA). The expression levels of 16 genes were investigated, and quantitative real-time PCR assays (RT-qPCR) were performed with Fast Start Essential DNA green master (Roche, Mannheim, Germany). Primers were synthesized by Oligomer Biotechnology (Ankara, Turkey) and Macrogen (Seoul, South Korea). The sequences of primers are shown in Table 1. All data were normalized with the β-actin gene. The primers for these genes were designed using the Oligo 7 program (https://www.oligo.net, accessed on 12 July 2022) and Primer-Blast (https://www.ncbi.nlm.nih.gov/tools/primer-blast, accessed on 13 July 2022) tools.

### 4.7. Wound Healing Assay

The wound healing assay is performed to determine cell mobility. A549 cells were seeded in a 12-well plate and incubated until cell confluence reached about 90%. For the co-cultured A549 cells, 1.2 × 10^5^ monocytes/transwell were seeded in the 12-well transwell insert with a pore size of 0.4 mm. After the polarization of monocytes to M2 macrophages in co-culture conditions, the transwell inserts were placed onto A549 cells and both cells were incubated for 48 h in serum-free medium. Simultaneously, monocultured A549 cells were incubated for 48 h in serum-free medium. Next, straight scratches were made with a 200 mL pipette tip. The cells were washed with phosphate-buffered saline (PBS) and incubated in serum-free medium treated with/without 20 μM vinorelbine. Images were visualized at 0, 24, 48, and 72 h using an inverted microscope and quantified by ImageJ software (version 1.53, NIH, Bethesda, MD, USA) [31]. Each experiment was performed in triplicate.

### 4.8. Western Blot Analysis

After A549 cells were treated with 50 μM of vinorelbine and incubated for 24 h and 48 h, the cells were washed with ice-cold PBS and then lysed using ice-cold RIPA lysis buffer. After that, the cell lysates were centrifuged at 12,000 rpm for 20 min at 4 °C and the supernatants were collected. The proteins were separated with 4 to 12% SDS gels and were transferred to a PVDF membrane. The membrane was blocked with fish gelatin blocking buffer for 1 h, then the membrane was incubated with the primary antibodies overnight at 4 °C. The dilution ratios of primers were used according to the manufacturer’s recommendations. The membrane was incubated with goat anti-mouse (1:5000) or goat anti-rabbit (1:10,000) HRP conjugate secondary antibodies (Advansta, San Jose, CA, USA) for 1 h. Finally, to detect the proteins, the membrane was visualized using an ECL Western blotting detection reagent. The primary antibodies used were a rabbit anti-EGFR antibody (Cat. No. cs-2232s) and a mouse anti-STAT3 antibody (Cat. No. cs-9139p). These were obtained from Cell Signaling Technology (Danvers, MA, ABD). Mouse anti-p-EGFR antibody (Cat. No. 943401) and mouse anti-b-actin antibody (Cat. No. 643802) were obtained from BioLegend (San Diego, CA, USA). Rabbit anti-p-STAT3 (Tyr 705) antibody (Cat. No. G0209) was bought from Santa Cruz Biotechnology (Dallas, TX, ABD). Mouse anti-BAX antibody (Cat. No. 556467) was bought from (BD Biosciences, Franklin Lakes, NJ, USA) [31,44].

### 4.9. Statistical Analysis

Statistical analysis was performed using GraphPad Prism 7 software (GraphPad, San Diego, CA, USA). Each experiment was performed in at least triplicate. The data in the assays are expressed as mean ± SD. The statistical evaluation of the data was performed using an one-way analysis of variance (ANOVA). Relative expressions of genes were determined with the 2-ΔΔCt method. *p* < 0.05 was considered statistically significant.

## Figures and Tables

**Table 1:** RT-qPCR primers used in this study.

Gene Name	Sequence
β-Actin	5′-ATGATGATATCGCCGCGCTC-3′ 5′-TCGTCGCCCACATAGGAATC-3′
MMP9	5′-CTGGAGGTTCGACGTGAAGG-3′ 5′-TCGGTACTGGAAGACGTCGTG-3′
CXCL8	5′-CCAGGAAGAAACCACCGGAA-3′ 5′-CGCAGTGTGGTCCACTCTCAA-3′
EGF	5′-CAGTGAAGTCAGCCAGAGCA-3′ 5′-TGGGGCATCTTTTACTCTCCT-3′
EGFR	5′-CTACAACCCCACCACGTACC-3′ 5′-GCCCTTCGCACTTCTTACACT-3′
PDGFA	5′-TTAAAAATCGGGGAGGGGAGT-3′ 5′-TCAGGCTGGTGTCCAAAGAA-3′
TGF-β1	5′-TGGACATCAACGGGTTCACT-3′ 5′-CTTGGGCTCGTGGATCCACTT-3′
TGFBR1	5′-TAACTGAGGTTAGAGCTAGTG-3′ 5′-AATGTAAGAAGACCATGACAA-3′
TGFBR2	5′-GACAATCACACATGCAGTGG-3′ 5′-AAGAGAAGTGCTAGGCAGGGA-3′
STAT3	5′-GGTGCCTGTGGGAAGAATCA-3′ 5′-GACATCCTGAAGGTGCTGCT-3′
FGF2	5′-GAAGAGCGACCCTCACATCA-3′ 5′-AGCCAGGTAACGGTTAGCAC-3′
PD-L1	5′-GGTGCCGACTACAAGCGAA-3′ 5′-TCTTGGAATTGGTGGTGGTGG-3′
CCL17	5′-ATTCAAAACCAGGGTGTCTC-3′ 5′-GGTACCACGTCTTCAGCTTTC-3′
CCL22	5′-CCTGGGCTGAGACATACAGGA-3′ 5′-CGGTAACGGACGTAATCACGG-3′
BCL2	5′-GAACTGGGGGAGGATTGTGG-3′ 5′-CCGTACAGTTCCACAAAGGC-3′
BAX	5′-GTCTTTTTCCGAGTGGCAGC-3′ 5′-GGGACATCAGTCGCTTCAGT-3′

## Data Availability

The original contributions presented in this study are included in the article. Further inquiries can be directed to the corresponding author(s).

## Abbreviations

A549	Adenocarcinomic human alveolar basal epithelial cells
NSCLC	Non-small-cell lung cancer
PBS	Phosphate-buffered saline
FBS	Fetal Bovine Serum
RT-qPCR	Reverse-Transcriptase Quantitative PCR
VNB	Vinorelbine
